# Ubiquitin-mediated proteasome degradation regulates optic fissure fusion

**DOI:** 10.1242/bio.044974

**Published:** 2019-06-12

**Authors:** Warlen Pereira Piedade, Sydney Veith, Jakub Konrad Famulski

**Affiliations:** University of Kentucky, Department of Biology, 40506, Lexington, KY, USA

**Keywords:** Optic fissure, SIAH, Nlz2, Proteasome, Pax2, Retina, Coloboma

## Abstract

Optic fissure fusion is a critical event during retinal development. Failure of fusion leads to coloboma, a potentially blinding congenital disorder. Pax2a is an essential regulator of optic fissure fusion and the target of numerous morphogenetic pathways. In our current study, we examined the negative regulator of *pax2a* expression, Nz2, and the mechanism modulating Nlz2 activity during optic fissure fusion. Upregulation of Nlz2 in zebrafish embryos resulted in downregulation of *pax2a* expression and fissure fusion failure. Conversely, upregulation of *pax2a* expression also led to fissure fusion failure suggesting Pax2 levels require modulation to ensure proper fusion. Interestingly, we discovered Nlz2 is a target of the E3 ubiquitin ligase Siah. We show that zebrafish *siah1* expression is regulated by Hedgehog signaling and that Siah1 can directly target Nlz2 for proteasomal degradation, in turn regulating the levels of *pax2a* mRNA. Finally, we show that both activation and inhibition of Siah activity leads to failure of optic fissure fusion dependent on ubiquitin-mediated proteasomal degradation of Nlz2. In conclusion, we outline a novel, proteasome-mediated degradation regulatory pathway involved in optic fissure fusion.

## Abstract (precis)

**Summary:** Optic fissure fusion, a key retinal morphogenic event highly sensitive to developmental signaling, is directly regulated by ubiquitin-mediated proteasomal degradation uncovering a novel regulatory pathway potentially correlated to incidence of coloboma.

## INTRODUCTION

Coloboma is a congenital blindness disorder occurring approximately once in every 2077 live births in the US (Nakamura et al., 2011). Worldwide, it accounts for approximately 10% of pediatric blindness which makes it the most common childhood blinding disorder currently lacking a cure (Babcock et al., 2014; Bernstein et al., 2018; Gregory-Evans et al., 2004; Wen et al., 2015). Although it is known that most colobomas result from failure of optic fissure (OF) fusion, due to its genetic heterogeneity, the molecular basis of this developmental defect for most patients remains unclear (Nakamura et al., 2011; Bernstein et al., 2018; Wen et al., 2015; Cai et al., 2014; Pillai-Kastoori et al., 2014).

OF fusion depends on several morphogenetic pathways, including retinoic acid (RA), Sonic hedgehog (Shh), Fibroblast growth factor (FGF), bone morphogenic protein (BMP) and Wnt signaling (Gregory-Evans et al., 2004; Cai et al., 2014). Mutations attributed to these pathways have been implicated in coloboma cases and helped to create a coloboma gene network (Gregory-Evans et al., 2004). Interestingly, many of these pathways converge in the precise timing of regulation of the paired–box (Pax) transcription factor Pax2. *Pax2* is expressed in the ventral optic cup, specifically the OF and optic stalk and is known to be essential for OF fusion (Macdonald and Wilson, 1996). Loss of pax2 function in mice, zebrafish and humans (heterozygous) results in OF fusion defects and ultimately coloboma (Gregory-Evans et al., 2004; Eccles and Schimmenti, 1999; Keller et al., 1994; Sanyanusin et al., 1995; Torres et al., 1995).

In 2009, Brown et al., used morpholinos to knockdown Nlz2, a zinc-finger transcription factor, and documented an increase in *pax2* gene expression in addition to failure of OF fusion (Brown et al., 2009). Furthermore, chromatin immunoprecipitation confirmed that Nlz2 was able to bind to a conserved segment of the *pax2* promoter. This finding suggested that regulation of *pax2* expression requires not only proper induction but also repression during OF fusion. We therefore sought to investigate how Nlz2 is regulated during retinal morphogenesis.

Fine-scale control of quantitative-spatiotemporal gene expression patterns is often accomplished by combinatorial regulatory events while the final control checkpoint often depends on post-translational modifications (Bhattacharjee et al., 2013). The ubiquitin-proteasomal system (UPS) is one example of post-translational modification which is well known to play a pivotal role in regulating protein activity, stability, and function in order to fine-tune gene expression during development (Bhattacharjee et al., 2013; Yao and Ndoja, 2012). The Siah family of E3 ubiquitin ligases are members of the UPS system and are known to play a role in retinal development (Bogdan et al., 2001; Hu et al., 1997). Siah is a vertebrate homologue of *Drosophila* seven in absentia, a regulator of *Drosophila* photoreceptor development, in particular specification of the R7 photoreceptor (Carthew and Rubin, 1990). In addition, siah activity is known to be involved in vertebrate axis formation, hypoxia signaling, DNA damage and cellular senescence (Ekker et al., 1995; Kang et al., 2014; Qi et al., 2013). Siah, binds to substrates and targets them for proteasome-mediated degradation upon recognizing a common and conserved binding motif that acts as a degradation signal or ‘degron’: P-[ARTE]-x-V-x-P, with the core V-x-P constituting residues with highest conservation (House et al., 2003). Zebrafish express two members of this family, s*iah1* and *siah2l*, both of which are predicted to recognize the same degron motif (House et al., 2006). Interestingly, the Nlz2 coding sequence contains an evolutionarily conserved siah degron motif suggesting it is subject to regulation via the UPS system during development (Table S1).

Our present study aimed to investigate the regulation of Nlz2 activity and ultimately OF fusion by the UPS. Toward this goal our data indicate that Nlz2 function is subject to UPS regulation via the E3 ubiquitin ligase Siah1 and Hedgehog signaling. Ultimately, this pathway functions to regulate appropriate levels of pax2a mRNA during optic fissure fusion. By our understanding, this is the first example of the UPS system playing a role in OF fusion and its connection to Hedgehog signaling.

## RESULTS

### Nlz2 is a negative regulator of *pax2a* gene expression

**Fig. 1.**
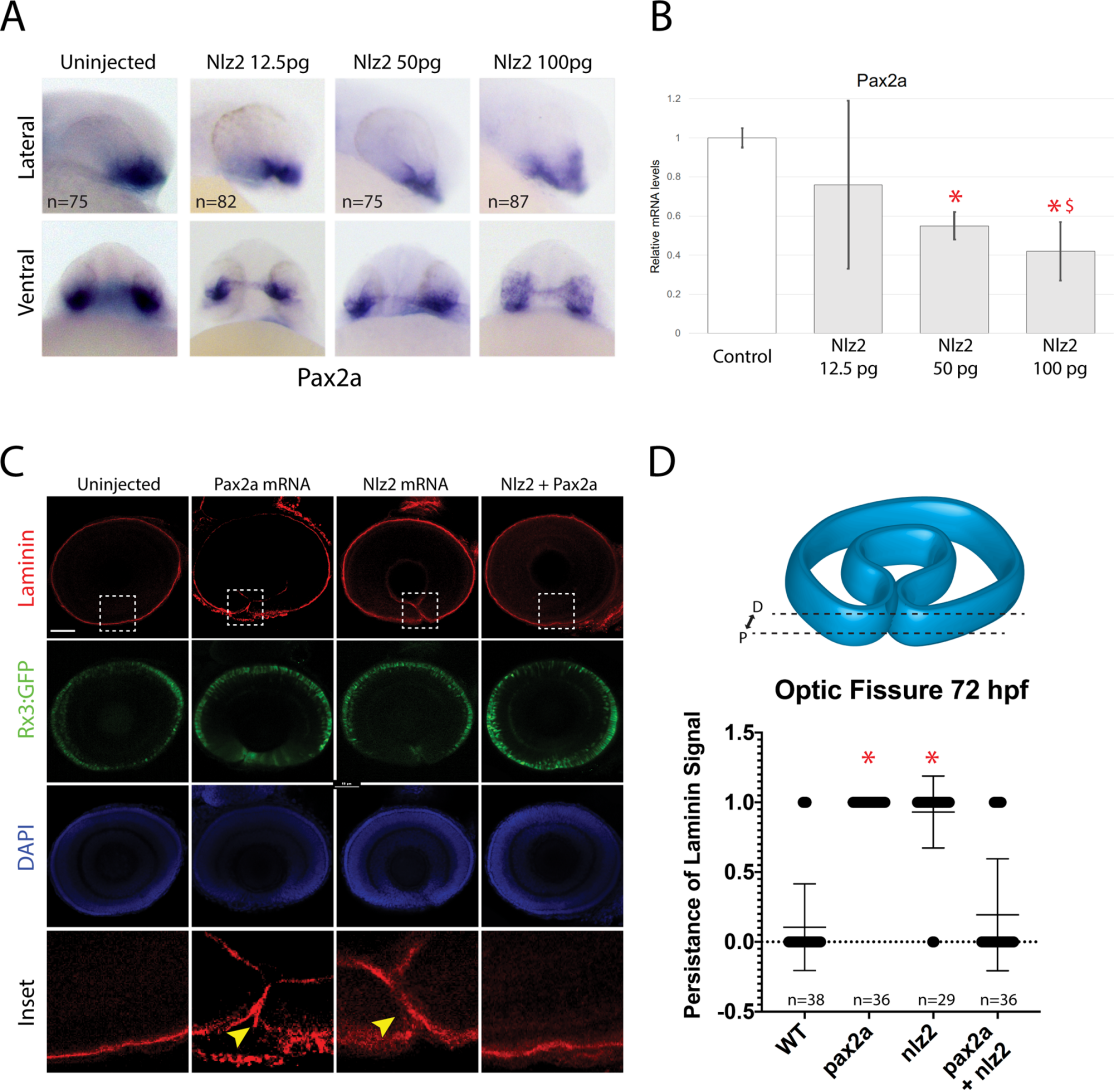
**Nlz2 regulates optic fissure closure by inhibiting *pax2a* gene expression.** (A) WISH for *pax2a* in 24 hpf embryos injected with varying amounts of Nlz2 mRNA. OF-associated *pax2a* signal is decreased in response to increasing Nlz2 mRNA. (B) qPCR results at 24 hpf for *pax2a* expression±s.d. *P<0.05 compared to uninjected, ^$^*P*<0.05 compared to Nlz2 50 pg. (C) 72 hpf Tg[*rx3*:GFP] (green) embryos injected with Pax2a, Nlz2 or Pax2+Nlz2 mRNA stained for laminin (red) and DAPI (blue). Arrowheads indicate persistence of laminin signal. Scale bar: 50 µm. (D) Proportion of embryos with failure of fusion (1) or completed fusion (0)±s.d. Region of analysis is outlined by dashed lines. *P<0.05 compared to uninjected, one-way ANOVA; *P*<0.0001. Dashed boxes outline region of inset.

To confirm Nlz2 is a negative regulator of *pax2a* expression we injected Nlz2 mRNA into single-cell zebrafish embryos and subsequently analyzed *pax2a* expression. Whole-mount *in situ* hybridization (WISH) analysis of Nlz2 mRNA injections confirmed a dose-dependent reduction of *pax2a* expression (Fig. 1A) which was validated by qPCR (Fig. 1B). Pax2 is an essential regulator of optic fissure fusion. We therefore sought to determine whether Nlz2-dependent modulation of *pax2a* expression levels would have consequences on optic fissure fusion. Optic fissure fusion in embryos injected with Pax2a, Nlz2 or both mRNAs was analyzed by examining the degradation of basement membrane (BM) as indicated by laminin immunohistochemical (IHC) staining (Fig. 1C). At 72 hpf, when fissure fusion is largely completed in zebrafish, we observed persistence of laminin in the fissure in both Pax2 and Nlz2 injected embryos but not in controls (Bernstein et al., 2018; James et al., 2016). This suggests that both an increase (Pax2 mRNA injected) or decrease (Nlz2 mRNA injected) of *pax2a* gene expression has consequences on OF fusion. Co-injection of both Pax2 and Nlz2 mRNA rescued OF fusion indicating that inhibition of *pax2a* expression by Nlz2 was compensated by the injected Pax2 mRNA to levels that were compatible with fissure fusion (Fig. 1C). Our data therefore support the previous findings where knockdown of Nlz2 resulted in fissure fusion failure, which we suggest is due to an increase in *pax2a* expression. It is well known that Pax2 loss of function is directly correlated to fissure fusion failure, however, our findings indicate that *pax2a* gene expression also requires negative regulatory control for ensuring appropriate levels of expression. We therefore propose that Nlz2 is a critical modulator of this process and sought out to understand how Nlz2 itself is regulated.

### Nlz2 is a target for proteasomal degradation

**Fig. 2.**
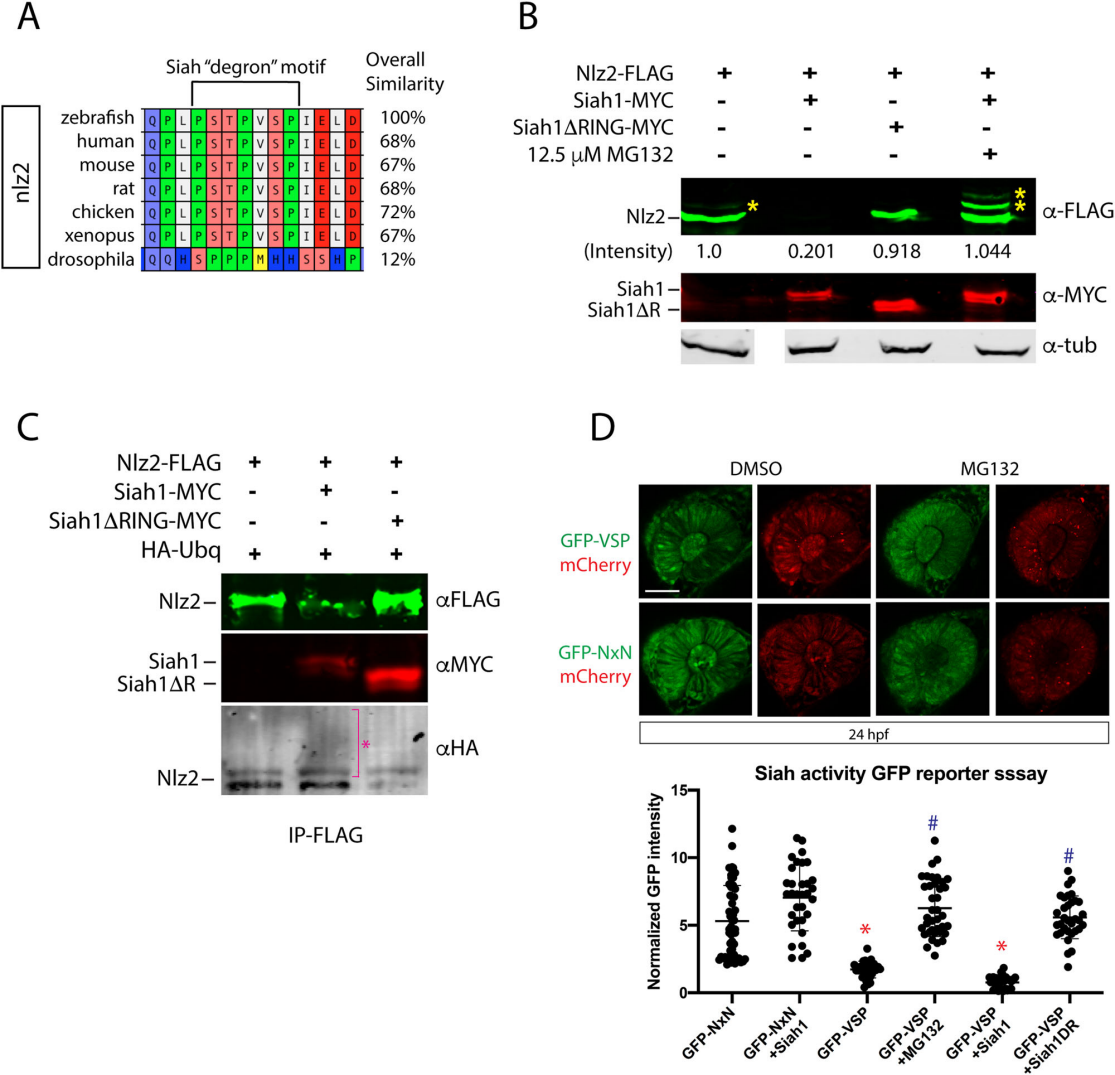
**Siah targets Nlz2 for proteasomal degradation.** (A) Conservation of Nlz2 ‘degron’ motif sequence. (B) Western blot analysis of nlz2-FLAG protein stability in response to siah1-myc, siah1ΔR-myc or siah1-myc+MG132. Actin was used as a loading control. * indicates potential ubiquitination products. Nlz2-FLAG band intensity quantification is shown. (C) Co-immunoprecipitation of nlz2-FLAG co-transfected with HA-ubiquitin, siah1-myc or siah1ΔR-myc probed for FLAG (green), MYC (red) and HA (B/W). * indicates potential ubiquitination. (D) Upper panels: endogenous Siah activity reporter assay expression in eyes of 24 hpf GFP-NxN or GFP-VSP mRNA-injected embryos, ±12.5 µM MG132. mCherry mRNA was co-injected for normalization. Scale bar: 50 µm. Lower panel: quantification of normalized GFP fluorescence intensity in the eye±s.d. *P<0.05 compared to GFP-NxN, ^#^*P*<0.05 compared to GFP-VSP, one-way ANOVA; *P*<0.0001.

Proteasomal degradation is a well-characterized mechanism for regulating protein levels which in turn enables precise modulation of biological activity. Siah1 and 2 have both been shown to target a conserved degron motif: Px[ARTE]xVxP. A search of the zebrafish proteome for this degron sequence revealed 70 potential targets, one of which was found to be highly conserved in the sequence of Nlz2 (Fig. 2A, Table S1). To determine whether Siah can directly target Nlz2 for proteasomal degradation, HEK293 cells were co-transfected with nlz2-FLAG, siah1-myc or siah1ΔR-myc, a deletion of the RING domain which has been previously shown to not only inactivate Siah, but also act as a dominant negative, and analyzed via western blot (Fig. 2B) (House et al., 2006). The results clearly show a significant reduction of nlz2-FLAG, down to 20%, upon co-expression with siah1-myc but not with siah1ΔR-myc or upon treatment with a proteasome inhibitor MG132 (Fig. 2B). Interestingly, HEK293 cells appear to have minor endogenous Siah activity as observed by the faint mono-ubiquitinated band of Nlz2-FLAG. Additional and much stronger bands are observed when Siah1-myc is co-expressed with Nlz2-FLAG (Fig. 2B). Furthermore, nlz2-FLAG was able to immunoprecipitate siah1-myc and displayed mono- as well as poly-ubiquitination when co-expressed with HA-ubiquitin (Fig. 2C). Nlz2-FLAG mono- and poly-ubiquitination appears greatly reduced when co-expressed with siah1ΔR-myc (Fig. 2C). We therefore conclude that Nlz2 is a direct target of Siah1 and subject to proteasomal degradation. Our finding therefore support the idea that Siah1 modulates Nlz2 activity in order to indirectly modulate levels of Pax2a mRNA during OF fusion.

Having shown Siah1 can directly target Nlz2 for proteasomal-mediated degradation, we next determined whether Siah1 is catalytically active during zebrafish retinal morphogenesis. To do so, we employed a GFP reporter assay shown previously to detect Siah activity *in vivo* (Famulski et al., 2010). In order for GFP fluorescence to become a readout of zebrafish endogenous Siah activity, we fused the Nlz2 degron motif to the C-terminus of GFP (GFP-VSP) and injected mRNA into 1-cell embryos. As a control, we also generated a GFP-degron fusion with a mutation that renders the degron desensitized to siah (GFP-NxN). At 24 hpf, fluorescence signal in the developing eye was significantly reduced in GFP-VSP-injected embryos compared to GFP-NxN controls, indicating Siah is active during early eye development (Fig. 2C). When examining distribution of the GFP-VSP signal we did not detect any regional concentration of siah activity, rather the entire retina appeared to contain active Siah. As control, treatment with MG132 restored GFP-VSP signal throughout the eye, while co-injecting Siah1 mRNA further reduced GFP-VSP signal. Furthermore, Siah1 co-injection had no effect on GFP-NxN signal. Lastly, co-injection with Siah1ΔR led to an increase of GFP-VSP signal due to inhibition of endogenous Siah activity (Fig. 2D). Overall, our reporter assay indicates that Siah is in fact active during early zebrafish retinal morphogenesis and may therefore play a functional role in OF fusion.

### *siah1* and *siah2l* are expressed in the retina during early eye morphogenesis

**Fig. 3.**
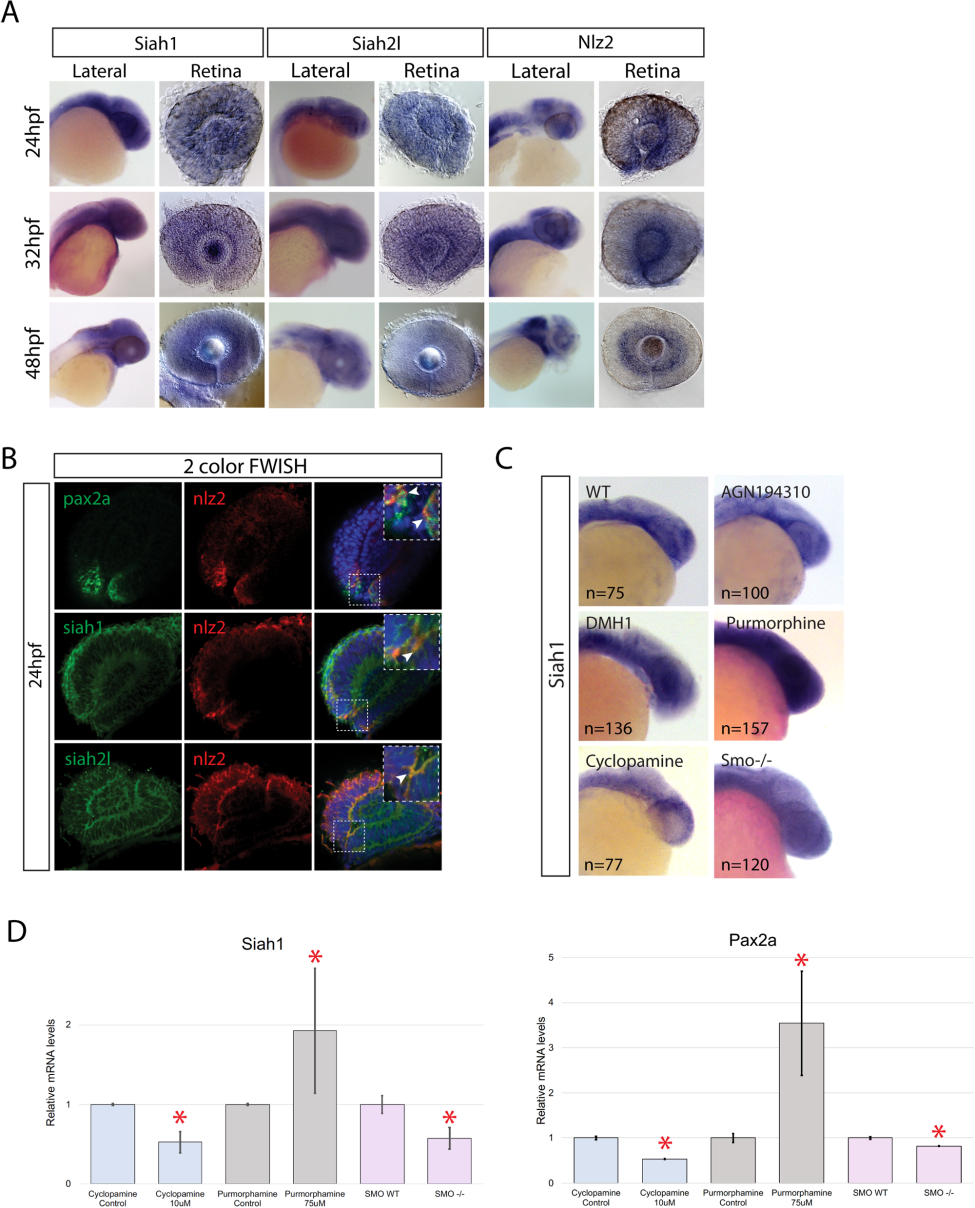
***siah1, siah2l* and *nlz2* gene expression during eye morphogenesis.** (A) WISH for *siah1*, *siah2l* and *nlz2* between 24 and 48 hpf. (B) Two-color fluorescent WISH (FWISH) for *nlz2*, *siah1*, *siah2* and *pax2a*. Arrowheads indicate co-localization. (C) s*iah1* WISH after treatment with RA (AGN194310), BMP (DMH1), hedgehog (cyclopamie) inhibitors, hedgehog agonist (purmorphamine) or in smo^hi1640Tg embryos at 24 hpf. (D) qPCR results for *siah1* and *pax2a* expression±s.d. *P<0.05 compared to respective controls.

Having confirmed Siah activity in the developing eye we next examined expression of *siah* and *nlz2* during early eye morphogenesis. WISH analysis between 24 and 48 hpf indicated that *siah1* and *siah2l* are both expressed homogeneously throughout the eye and central nervous system (Fig. 3A). WISH for *nlz2* confirmed expression in the margins of the optic fissure at 24 hpf (Fig. 3A). Interestingly, by 48 hpf *nlz2* expression appears to coincide with the first wave of newly differentiated retinal ganglion cells and no longer with the OF. In order to confirm co-expression between *nlz2*, *pax2a* and *siah* in the OF, we performed two-color fluorescent WISH (FWISH) (Fig. 3B). Confocal sections clearly indicate co-expression of *siah1* or *siah2l* and *nlz2*, as well as *nlz2* and *pax2a* in the optic fissure. All three components of our model are therefore co-expressed and amendable to interaction. To further investigate the regulation of *siah1* expression we inhibited the retinoic acid, BMP and Hedgehog signaling pathways pharmacologically (Fig. 3C). Using WISH, and confirmed by qPCR, we observed an upregulation of *siah1* expression upon inhibition of BMP signaling and a decrease upon inhibition of hedgehog signaling (Fig. 3D). There was no difference upon inhibition of RA signaling. BMP signaling is known to antagonize Shh during early retinal morphogenesis (French et al., 2009), as such, Shh appears to be a regulator of *siah1* expression. In fact, upregulation of hedgehog signaling (purmorphamine treatment) also resulted in an upregulation of *siah1* expression while smo^hi1640Tg mutant embryos exhibited a decrease of *siah1* expression (Fig. 3C,D). Hedgehog signaling is known to be essential for proper OF fusion and also *pax2a* expression (Morcillo et al., 2006). We verify this fact in our treatments by analyzing *pax2a* expression via qPCR (Fig. 3D). Our data indicate that inhibition of hedgehog leads to a decrease in *pax2a* expression while activation of hedgehog leads to an increase. Based on our findings, we propose that Shh may indirectly regulate levels of *pax2a* expression by maintaining *siah1* expression, and therefore modulate activity Nlz2 during OF fusion.

### Siah activity is required during embryonic development

To test our model, we used mRNA injections to regulate the activity of Siah. Siah1 and Siah2l mRNA was used to upregulate siah activity while Siah1ΔR or Siah2lΔR mRNA served to inhibit endogenous Siah. Injection of Siah1 or Siah2l mRNA had morphological consequences on early embryo morphogenesis in a dose-dependent manner. As did injections of siah1ΔR or siah2lΔR. In general, we classified 24 hpf injected embryos into four types, WT (I), mild morphological defects (II), posterior defects (III) and anterior defects (IV) (Fig. S1). Siah1 or Siah2l injections both resulted in a ∼50% split between type I and types II, III and IV. Results of our injections were similar to previous attempts at modulating Siah activity in zebrafish (Kang et al., 2014). For our analysis of retinal morphogenesis we exclusively examined type I embryos. In order to ensure the phenotypes observed were dependent on siah activity, we also treated Siah1 or Siah2l-injected embryos with MG132 (Fig. S1). Inhibition of the proteasome reduced the incidence of type II, III and IV embryos in both cases, indicating that our mRNA induced phenotypes resulted from Siah activity. Our results indicate that Siah activity is required for multiple steps during early development and its levels need to be carefully controlled.

### Siah indirectly regulates *pax2a* gene expression and optic fissure closure

**Fig. 4.**
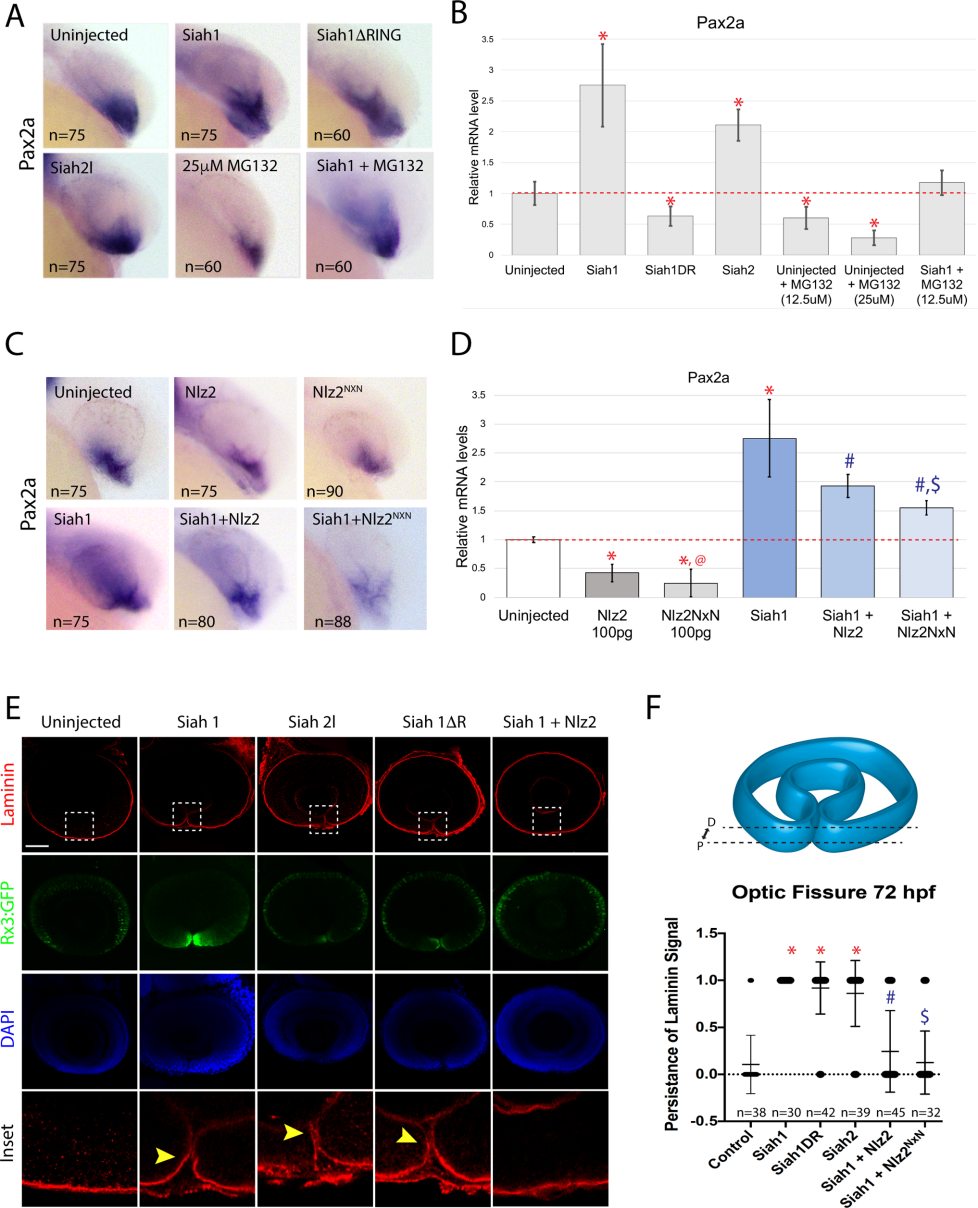
**Siah activity indirectly regulates *pax2a* expression.** (A) WISH for *pax2a* after injection of Siah1, Siah1ΔR, Siah2l or Siah2lΔR±MG132 at 24 hpf. (B) qPCR results for *pax2a* expression at 24 hpf. *P<0.05 compared to uninjected. (C) WISH for *pax2a* gene expression upon co-injection of Siah1+Nlz2 or Nlz2^nxn^. (D) qPCR results for *pax2a* expression at 24 hpf. *P<0.05 compared to uninjected, ^@^*P*<0.05 compared to Nlz2, ^#^*P*<0.05 compared to Siah1, ^$^*P*<0.05 compared to Siah1+Nlz2. (E) Tg[*rx3*:GFP] (green) embryos injected with siah1, siah2l, siah1ΔR or siah2lΔR±100 pg Nlz2 mRNA stained for laminin (red) and DAPI (blue) at 72 hpf. Arrowheads indicate persistence of laminin. Scale bar: 50 µm. (F) Proportion of embryos with failure of fusion (1) or completed fusion (0). Region of analysis is outlined by dashed lines. *P<0.05 compared to uninjected, ^#^*P*<0.05 compared to Siah1, ^$^*P*<0.05 compared to Siah1+Nlz2. One-way ANOVA; *P*<0.0001. Dashed boxes indicate region of inset.

Ultimately, our model predicts that Siah activity will indirectly control the levels of *pax2a* gene expression. As such, we sought out to analyze *pax2a* gene expression upon modulation of Siah activity. Embryos injected with Siah mRNA constructs were subsequently analyzed for *pax2a* expression using WISH. At 24 hpf, using WISH and qPCR, we observed an increase in *pax2a* expression upon activation of siah activity, Siah1 or Siah2l mRNA injection (Fig. 4A) which was validated using qPCR (Fig. 4B). This fit our model where an increase in Siah would result in a decrease in Nlz2 protein activity and therefore result in an increase of *pax2a* mRNA. Conversely, when we analyzed embryos injected with our ΔR constructs *pax2a* expression decreased (Fig. 4A,B). These findings also fit our model because decreased Siah activity would increase Nlz2 protein levels and in turn down regulate *pax2a* expression. Furthermore, proteasome inhibition (MG132) in control and Siah1 injected embryos had the expected effect of decreasing *pax2a* mRNA (Fig. 4A,B). To go one step further, we also constructed a Siah-desensitized Nlz2 variant by changing the core degron motif components from VxP to NxN (Fig. S2). When injected into controls or co-injected with Siah1, Nlz2^nxn^ resulted in a further decrease of *pax2a* mRNA compared to WT Nlz2 (Fig. 4C,D). Taken together, we conclude that Siah can indirectly modulate *pax2a* expression via regulation of Nlz2 protein stability through the UPS system. We hypothesize that this mechanism ensures that levels of Pax2 remain optimal thus ensuring proper timing and progression of OF fusion.

As outlined in Fig. 1C, misregulation of *pax2a* expression leads to failure of OF fusion. We therefore analyzed the consequences of Siah-mediated regulation of *pax2a* expression on OF fusion. Embryos injected with Siah1, Siah2l or Siah1ΔR, Siah2lΔR were grown to 72 hpf and analyzed for status of fissure fusion by laminin IHC (Fig. 4E). When Siah activity was upregulated, (Siah1 or Siah2l mRNA injection) we observed a persistence of laminin in the fissure at 72 hpf in more than 75% of embryos (Fig. 4F). We also observed similar results when inhibiting Siah activity upon injection of our ΔR constructs. Both conditions, as shown previously, misregulate levels of Pax2a mRNA. Co-injection Siah1 and Nlz2 resulted in a rescue of fissure fusion in 77.7% of embryos suggesting that increased levels of *nlz2* mRNA compensated for the increase in Siah activity (Fig. 4F). Furthermore, co-injection of Siah1+Nlz2^nxn^ mRNA resulted in an even higher rate of fusion at 87.5% (Fig. 4F). Our data therefore confirm that Siah activity can modulate OF fusion events by indirectly regulating *pax2a* mRNA levels through Nlz2 protein stability.

In conclusion, we have characterized a hedgehog-induced ubiquitin-mediated proteasome degradation pathway responsible for regulating *pax2a* expression during OF fusion. In our model (Fig. S3), Shh induces expression of the E3 ubiquitin ligase *siah1* which in turn regulates the levels of Nlz2 protein in the fissure margins to ultimately control *pax2a* mRNA levels. As development proceeds, Nlz2-mediated inhibition of *pax2a* prevents over-activation of Pax2a-mediated pathways in order to precisely time the initiation of fissure fusion (Fig. S3). By the time fusion is actively occurring at 48 hpf, *nlz2* is no longer expressed in the fissure and the window of *pax2a* regulation is therefore likely over. These findings open up new avenues of investigation in regard to coloboma etiology where negative regulation of *pax2a* expression as well as regulation of the UPS pathway present as new intriguing candidates for clinical examination.

## MATERIALS AND METHODS

### Zebrafish and embryo maintenance

Zebrafish were maintained using husbandry procedures approved by University of Kentucky IACUC committee. AB strains were used as wild type, Tg[*rx3*:GFP] (Famulski et al., 2010) transgenic line was use to visualize retinal morphogenesis. Smoothened mutant line smo^hi1640Tg was provided by Dr Kwan (University of Utah, USA). Embryos were kept at 28°C in E3 embryo media. Sex of individual embryos in our study could not be determined at the chosen developmental stages.

### Inhibitor treatments

Embryos were incubated in embryo media with DMSO or: 12.5 µM or 25 µM of MG132 (Sigma-Aldrich) in DMSO, 10 µM AGN 194310 (Med Chem Express) in DMSO, 0.2 µM DMH1 (Sigma-Aldrich) in DMSO, 10 µM cyclopamine (Sigma-Aldrich) in EtOH or 75 µM purmorphamine (Sigma-Aldrich) in DMSO starting at 5.5 hpf for cyclopamine and purmorphomine and 10 hpf for AGN194310, DMH1 and MG132, all embryos were fixed at 24 hpf.

### Whole-mount *in situ* hybridization

WISH was performed as previously described (Holly et al., 2014). RNA probes were generated using PCR with T7 promoter sequence and subsequently transcribed (DIG or FITC labeled) using T7 polymerase (Roche). Primer sequences can be found in Table S2. Images were captured using a Nikon Digital sight DS-U3 camera and Elements software. Dissected eyes from 24, 32 and 48 hpf, embryos were mounted in 70% glycerol and imaged under DIC using a Nikon TiE compound microscope equipped with a 20× (0.95NA) objective and Elements software. Image adjustment was performed using Adobe Photoshop.

### Two-color fluorescent WISH

WISH protocol was performed as previously described (Holly et al., 2014). DIG and FITC probes were detected using FastBlue (Sigma-Aldrich) and FastRed (Sigma-Aldrich), respectively. Staining protocols were followed according to Lauter et al. (2011). Fluorescent images were collected using confocal microscopy.

### Immunofluorescence

For Laminin IHC, decorionated embryos were fixed with 4% PFA in PBS at room temperature for 3 h and washed with PBST three times for 10 min. Embryos were then permeabilized with Proteinase K, 30 µg/ml 15 min for 24 hpf, 50 µg/ml 30 min for 48 hpf and 75 µg/ml 30 min for 72 hpf, washed three times in PBST for 5 min and blocked overnight at 4°C with 10% sheep serum, 0.8% Triton X-100 and 1% BSA in PBS. Primary rabbit anti-laminin antibody (Thermo Fisher Scientific – 1:100) in blocking buffer (1% sheep serum, 1% BSA and 0.8% Triton X-100 in PBS) was incubated overnight at 4°C and washed five times in PBST for 10 min. Secondary antibodies, donkey anti-rabbit (Alexa Fluor^®^ 555 – Abcam – 1:1000) or goat anti-GFP Dylight 488 (Rockland – 1:500) and DAPI 1:1000, were incubated for 1 h at room temperature in the dark. The embryos were washed five times in PBST for 10 min and visualized.

### Confocal microscopy

Two-color FWISH, laminin IHC staining as well as GFP mRNA-injected embryos were imaged using a Nikon C2+ confocal microscope equipped with a 20× (0.95NA) water immersion objective. Embryos were embedded in 1% low melting point agarose on glass bottom 35 mm dishes (Fluoro dish, Precision Instruments). Images were captured using Nikon Elements software and manipulated using Adobe Photoshop.

### Endogenous Siah activity GFP assay

Embryos were co-injected with 50 pg GFP-VSP or GFP-NXN mRNA and 50 pg mCherry mRNA as a normalizing control. At 24 hpf, single confocal sections were collected form the central-distal region of the eye. Fiji software (https://fiji.sc) was used to outline and measure the fluoresce intensity of GFP signal observed in the eye of the embryo at 24 hpf. Intensity of the mCherry signal was used to normalize the intensity measured from GFP-VSP or GFP-NXN.

### Cloning and mRNA synthesis

Full coding domain sequences for Siah1 (Ensembl transcript ID: ENSDARG00000030871), Siah2l (Ensembl transcript ID: ENSDARG00000044381), Nlz2 (Ensembl transcript ID: ENSDARG00000018492) and Pax2a (Ensembl transcript ID: ENSDARG00000028148) were amplified and cloned into pCS2+. Siah1ΔRING and Siah2lΔRING were generated using overlapping PCR and cloned into pCS2+. GFP-VSP and NxN constructs were amplified from pCS2-EGFP and cloned into pCS2+. MYC tag was fused to the C-teminus of siah1 and siah1DR using PCR and subsequently cloned into pCIG2 (Famulski et al., 2010). FLAG tag was fused to the C-terminus of nlz2 using PCR and subsequently cloned into pCIG2. Primer sequences can be found in Table S2. All constructs were verified by sanger sequencing (Eurofinsgenomics). mRNA was synthesized from linearized pCS2 constructs using SP6 mMessage mMachine kit (Ambion) and purified using YM-50 Microcon columns (Amicon, Millipore).

### mRNA injections

mRNA was synthesized using the mMessage Machine SP6 kit (Ambion) and quantified using the Epoch Microplate Spectrophotometer (BioTek Instruments). Embryos were injected with 1–2 pl of diluted mRNAs. Unless indicated in figures, embryos were injected with: 100 pg pax2, 100 pg nlz2 or nlz2^nxn^, 300 pg of siah1, 500 pg of siah2l, 300 pg of siah1ΔR or 500 pg of Siah2lΔR.

### Real-time quantitative PCR

Primers for Pax2a and the endogenous control (GAPDH) were designed using Primer3Plus (https://primer3plus.com/cgi-bin/dev/primer3plus.cgi) and validated. Primer sequences can be found in Table S2. Total RNA was isolated from batches of 25–100 embryos at 24 hpf using TRIzol (Invitrogen). At least three independent biological replicates were performed for each treatment. cDNA was synthesized with 1 µg of total RNA using SuperScript reverse transcriptase (Invitrogen). cDNA was quantified using an Epoch Microplate Spectrophotometer (BioTek Instruments) and diluted to 100 ng/µl. Quantitative PCR was performed in triplicates using iTaq™ Universal SYBR^®^ Green Supermix (Bio-Rad), total reaction of 10 µl on a CFX Connect Real Time System (Boi-Rad). Melting curve analysis was done to determine the specificity of the primers. The results were analyzed using the standard ΔΔCt method. GAPDH was used as a reference gene in all analyses. Statistical significance was calculated using Microsoft Excel.

### Transfections and western blotting

HEK 293 cells were transfected using TransIT^®^-LT1 Transfection Reagent (Mirrus) at 37°C for 24 h with the following combinations: Nlz2:FLAG pCIG2 (1 µg); Siah1:MYC pCIG2 (2 µg)+Nlz2:FLAG pCIG2 (1 µg) and Siah1ΔRING:MYC (2 µg)+Nlz2:FLAG pCIG2 (1 µg). A fourth combination repeated Siah1:MYC pCIG2 (2 µg)+Nlz2:FLAG pCIG2 (1 µg) along with a treatment using 10 mM of MG132 in the last 4 h. Protein extraction was performed by adding Laemmli sample buffer (Sigma-Aldrich) and boiling the samples at 95°C for 10 min. Equal volume for each transfection was load on 10% SDS-PAGE and run for 90 min at 120 V. Then, they were transfer to a PVDF membrane for 90 min at 400 mA. The membrane was blocked overnight at 4°C using Odyssey blocking buffer (Li-COR). The membrane was incubated with rabbit anti-MYC (Sigma-Aldrich, 1:750) and mouse anti-FLAG (Sigma-Aldrich, 1:750) in 50% blocking buffer/PBS for 3 h at room temperature and immediately washed in five times PBST for 5 min. For detection of HA, rabbit anti-HA (Santa Cruz 1:500) was used. Subsequently, the membrane was incubated with goat 1:10,000 anti-mouse 800 (Rockland) and 1:10,000 goat anti-rabbit 700 (Rockland) in 50% blocking buffer/PBS containing 0.1% tween and 0.01% for 1 h at room temperature and immediately washed in three times PBST for 5 min. Two extra 5 min PBS wash steps were added to remove any Tween 20 residues. Rabbit anti β-tubulin (1:500 Cell Signaling Technology) was used as a loading control. Blots were scanned using Li-COR Odyssey infra-red imaging system. Band intensity was measured as pixel intensity from each scanned channel using ImageJ and normalized to the β−tubulin loading control.

### Co-immunoprecipitation

HEK 293 cells were transfected using TransIT^®^-LT1 Transfection Reagent (Mirrus) at 37°C for 24 h with the following combinations: Nlz2-FLAG pCIG2 (5 µg)+Ubiquitin-HA (1 µg); Siah1-MYC pCIG2 (5 µg)+Nlz2-FLAG pCIG2 (5 µg)+Ubiquitin-HA (1µg); Siah1ΔRING-MYC (5 µg)+Nlz2-FLAG pCIG2 (5 µg)+Ubiquitin:HA (1 µg). The cells were lysed in 100 µl of TCL buffer containing 50 mM Tris pH 7.5, 150 mM NaCl, 1% Triton, 1 mM Na_3_VO_4_, 10 mM NaF, PMSF, Aprotinin, Leupeptin, 1% SDS and 10 µM MG-132 boiled at 95°C for 10 min. 900 µl of TCL buffer without SDS was added to each sample and centrifuged for 10 min at 13,000 rpm. Protein concentrations were determined by Bradford assay and an equal amount of protein (1000 µg) were added to 40 µl of a 2:1 mixture of sepharose beads CL 4-B (Sigma-Aldrich, CL-4B-200) to Protein A sepharose CL-4B (GE Healthcare, 17-0780-01). 1 µg of mouse anti-FLAG (Sigma-Aldrich) antibody was added to the mix and incubated overnight at 4°C in shaker. Beads were collected by centrifugation and washed with 500 µl of TCL buffer three times for 5 min at 0.5 g and the immunocomplexes were eluted in 24 µl of Laemmli Sample Buffer (Sigma-Aldrich) and incubated at 95°C for 5 min. The immunocomplexes were then subjected to western blot analysis using a combination of rabbit anti-MYC (Sigma-Aldrich, 1:750) and mouse anti-FLAG (Sigma-Aldrich 1:750) or rabbit anti-HA (Santa Cruz, sc-805 – 1:1000) in 5% milk in TBS-T. Blots were scanned using Li-COR Odyssey infra-red imaging system.

### Statistical analysis

Two-factor analysis was done by unpaired Students *t*-test using GraphPad (https://www.graphpad.com). Data are shown as mean±s.d. By conventional criteria, a *P*-value of less than 0.05 was considered significant. ANOVA analysis was performed using Prism8.

## Supplementary Material

Supplementary information
