# Supplementary material for: Ubiquitin-mediated proteasome degradation regulates optic fissure fusion
Source: Biol Open. 2019 Jun 12;8(6):bio044974. doi: 10.1242/bio.044974 (PMC6602337; doi:10.1242/bio.044974)
Supplement: Supplementary information [file biolopen-8-044974-s1.pdf]

A

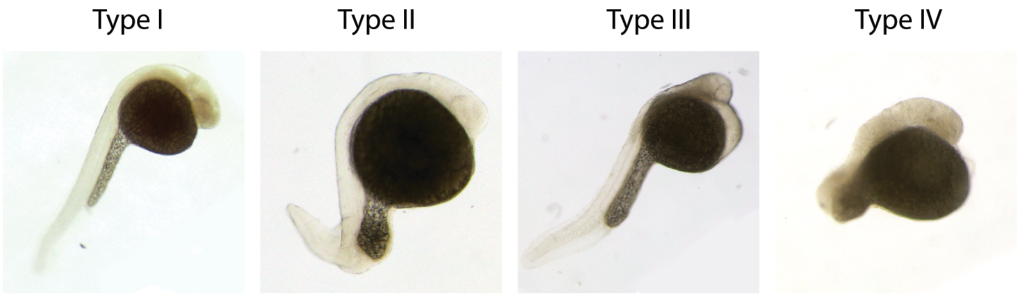

B

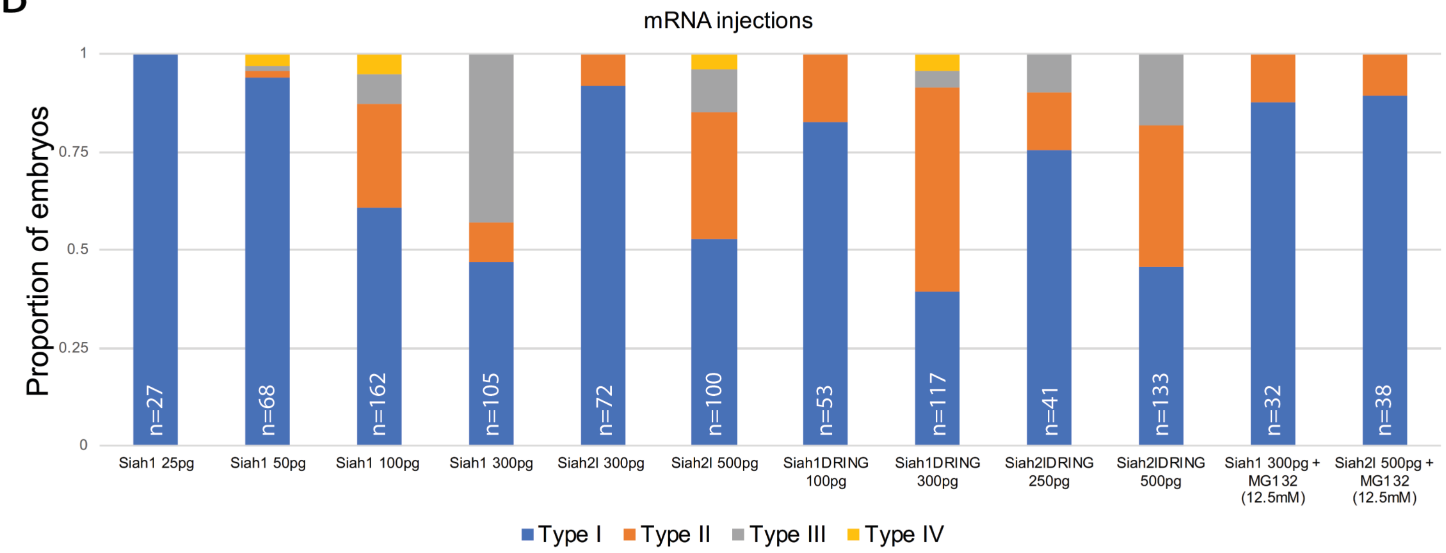

Figure S1: Siah gain and loss of function modulates zebrafish development. (A) Siah1 or Siah2l mRNA injected embryos were categorized into four groups. Type I: morphologically indistinct from controls. Type II: mild morphological defects, Type III: posterior defects, Type IV: anterior defects. Both siah1 and siah2l, as well as the dominant negative ΔR constructs induce morphological defects during zebrafish development. **(B)** Proportion of each phenotypic group is shown in the graph. The incidence of type II embryos was dose-dependent and rescued by MG132 treatment.

Biology Open • Supplementary information

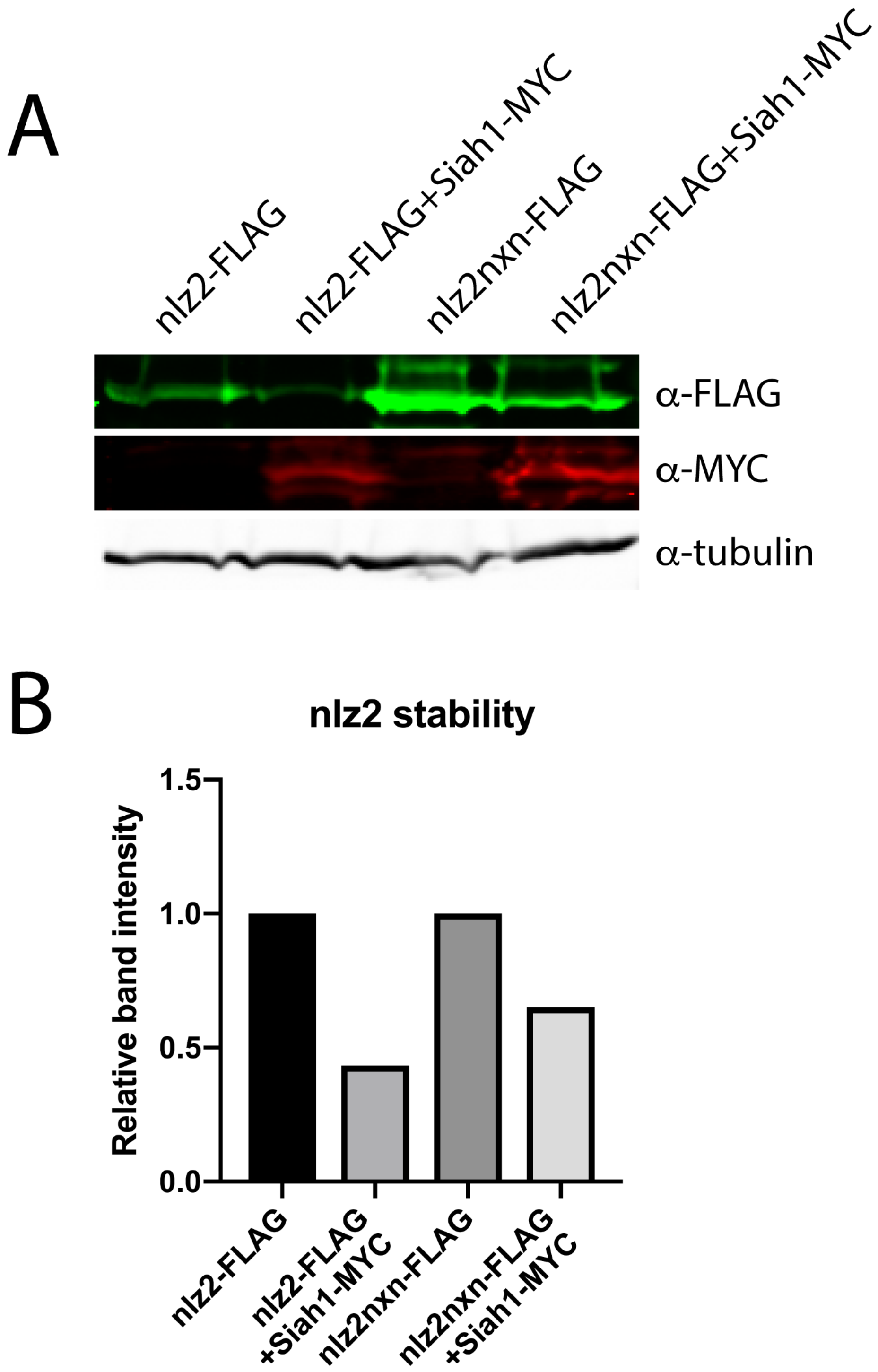

Figure S2: Response of nlz2^{nxn} to Siah activity.

A) Western blot analysis of nlz2-FLAG and nlz2^{nxn}-FLAG stability in the presence of Siah1-myc. Blots were probed for FLAG (green) and MYC (red). B-tubulin was used as a loading control. **B)** Quantification of nlz2-FLAG and nlz2^{nxn}-FLAG band intensity. Nlz2-FLAG is compared to nlz2-FLAG + siah1-myc while nlz2^{nxn}-FLAG is compared to nlz2^{nxn}-FLAG + siah1-myc.

Biology Open • Supplementary information

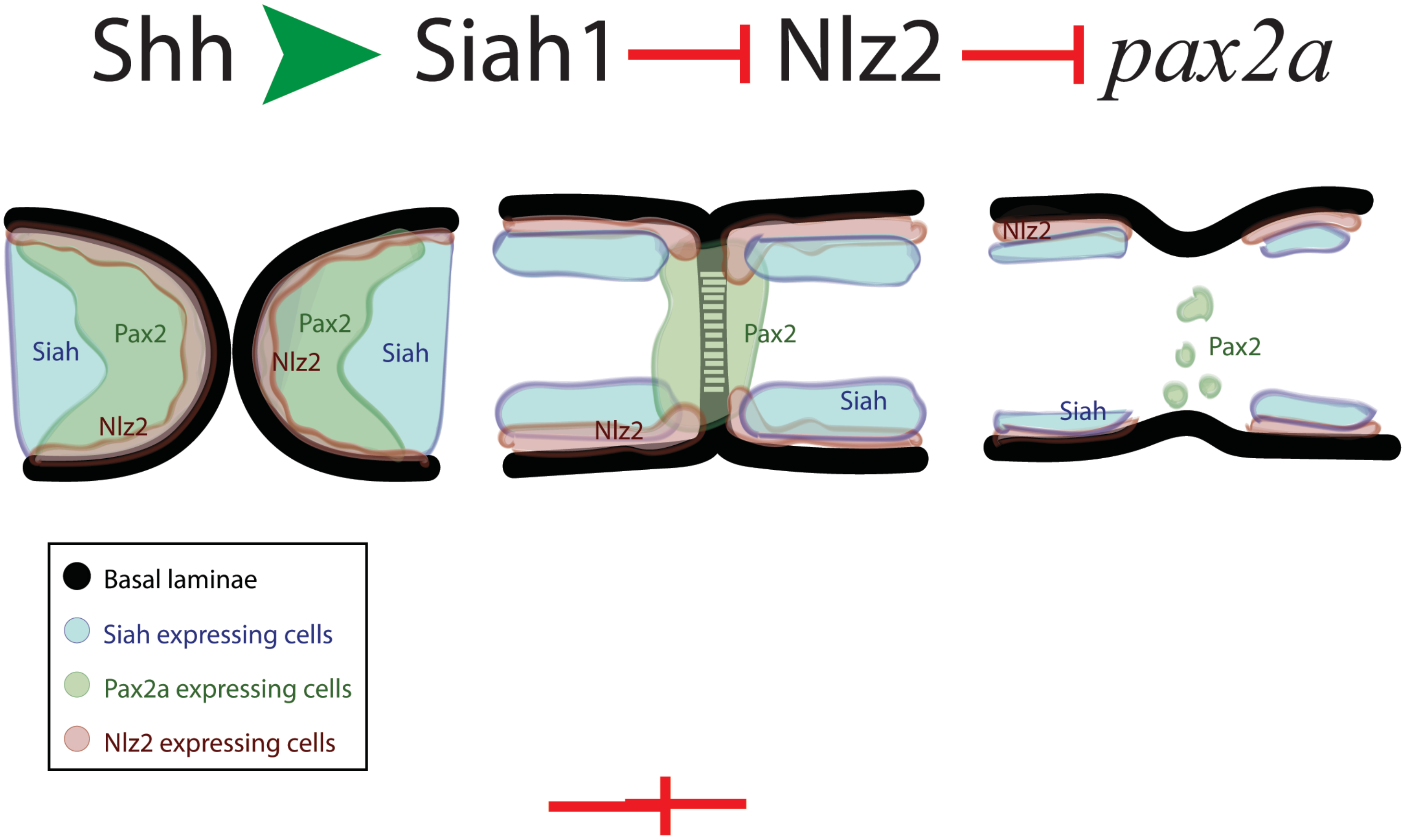

Figure S3: Model

Co-expression of nlz2, pax2a and siah maintains proper levels of pax2a mRNA to prevent premature activation of fusion. As development proceeds, nlz2 expression shifts from the border of the fissure and no longer regulates pax2a mRNA levels. At this point fissure fusion is already initiated and nearing its completion.

Biology Open • Supplementary information

Table S1: Siah degron containing zebrafish genes

Gene	Amino Acid	Degron
ADRM1	227 - 233	PtTpVtP
AL8A1	395 - 401	PvTcVtP
ARI5B	506 - 512	PpErVeP
ASAH2	605 - 611	PvAdVaP
AURKB	15 - 21	PsAgVgP
BMI1A	272 - 278	PsTlVqP
BSH	32 - 38	PlReVfP
CBPC5	422 - 428	PsEqVpP
CC70B	145 - 151	PqAhVeP
CDHR1	791 - 797	PnApVmP
CF165	317 - 323	PtThVfP
CHMP7	443 - 449	PrEsVsP
CHRD1	69 - 75	PaEpVkP
CMTR1	777 - 783	PaTsVaP
CYFP1	548 - 554	PrRaVgP
CYTSA	891 - 897	PaAaVsP
	997 - 1003	PtAsVnP
DCA17	119 - 125	PlEnVlP
DCR1B	316-322	PkAvViP
DUSTY	194 - 200	PcRnVqP
E4F1	66 - 72	PqTnVvP
FIGN	237 - 243	PqTpVaP
FUT11	421 - 427	PvEnVeP
GTDC1	359 - 365	PkAlVyP
GTPB3	272 - 278	PaAiVsP
HXB6A	128 - 134	PsApVyP
HXB6B	125 - 131	PcTpVyP
INS	51 - 57	PkRdVeP
INT12	111 - 117	PgEgVeP
ISPD	68 - 74	PvAaVlP
K0907	355 - 361	PvApVaP
	392 - 398	PtApVpP
MCA3A	1434 - 1440	PrTpVsP
MCA3B	1332 - 1338	PdRvVaP
MED12	1740 - 1746	PlTpVpP
MED6	213 - 219	PvEtVkP
MIO	507 - 513	PdTdVeP
MTMR2	217 - 223	PaTlVvP
MVP	379 - 385	PiEyVpP
MYSM1	206 - 212	PeRsVsP
NDOR1	456 - 462	PgTgVaP
NIPLA	442 - 448	PqEgVtP
OLA1	256 - 262	PgAlViP
PGAM5	180 - 186	PiEpVpP
PHC2	543 - 549	PqAvVkP
PHF2	910 – 916	PtArVgP
PHF8	838 - 844	PkArVtP
PLXA4	99 - 105	PpRiVqP
PREP	288 - 294	PnTaVpP
	565 - 571	PyTpVqP
PTC1	1169 - 1175	PpAeVvP
RBM42	76 - 82	PpTfVcP
	149 - 155	PmApVgP
RBM44	4-10.	PpAaVvP
RFIP3	249 - 255	PcEpVfP
RN185	73 - 79	PnRqVcP
RNF44	331 - 337	PpTaVgP
RTCB	107 - 113	PdAvVsP
SH34A	106 - 112	PsTyVqP
SH3R1	595 - 601	PtAaVtP
SPD2A	370 – 376	PeAgVaP
	673 - 679	PlRkVsP
SPT2	146 - 152	PsRpVkP
SPT6H	1244 - 1250	PeErVkP
STB1B	464 - 470	PtRpVaP
TBC23	524 - 530	PyRgVkP
TDRD5	520 - 526	PdAyVrP
TELO2	629 - 635	PiTaVtP
TEN3	1464 - 1470	PvRiVaP
TEX10	130 - 136:	PsErVaP
TRIO	2362 - 2368	PrAtVaP
	2368 - 2374	PlAlVkP
TTLL3	666 - 672	PhRlVlP
VWA8	1425 - 1431	PlAeVyP
WASH1	362 - 368	PsEvVqP
ZN503	77 - 83	PsTpVsP
ZNT6	424 - 430	PgRhVqP

Biology Open • Supplementary information

Table S2: Primers used

Primers		Primer sequence (5' to 3')
**WISH probes**	Siah1	Forward: ATGGACGAAGAAATGAGTCGC
		Reverse: TAATACGACTCACTATAGGGTCAGCACATAGATATGGTGAC
	Siah2l	Forward: ATGAGCCGTCCGTCCTCTGCG
		Reverse: TAATACGACTCACTATAGGGTTAGCACATAGAGATGGTCAC
	Nlz2	Forward: ATGATCACATCGCCCTCTGCT
		Reverse: TAATACGACTCACTATAGGGGCAGCCCAGTGAGCTGGCAG
	Pax2a	Forward: ATGGATATTCACTGCAAAGCAG
		Reverse: TAATACGACTCACTATAGGGCTAGTGGCGGTCATAGGCAGTG
	Siah1 sense	Forward: TAATTACGACTCACTATAGATGGACGAAGAAATGAGTCGC
		Reverse: GCACATAGATATGGTGACGTT
	Siah2l sense	Forward: TAATACGACTCACTATAGATGAGCCGTCCGTCCTCTGCG
		Reverse: TGATGGCCGCTGCAACACCGT
**Cloning**	Siah1 MYC	Forward: CATATCGATATGGACGAAGAAATGAGTCGC
		Reverse: ATGCTCGAGCTACAGGTCCTCCTCCGAGATCAACTTTTGTTCGCACATAGATATGGTGACGTTAATG
	Siah2l MYC	Forward: CATATCGATATGAGCCGTCCGTCCTCTGCG
		Reverse: ATGCTCGAGTTACAGGTCCTCCTCCGAGATCAACTTTTGTTCGCACATAGAGATGGTCACGTTG
	Nlz2 FLAG	Forward: CATATCGATATGATCACATCGCCCTCTGCTTC
		Reverse: ATGCTCGAGTCACTTATCATCGTCGTCCTTGTAGTCCTGGTATCCAAGCGCTGATGCTG
	Pax2a	Forward: CATGGATCCATGGATATTCACTGCAAAGCA
		Reverse: ATGCTCGAGCTAGTGGCGGTCATAGGCAGTG
	Siah1ΔRING MYC	Forward1: CATATCGATATGGACGAAGAAATGAGTCGC
		Reverse1: TCCGACCCAAGCGGAAACAGGCTGGCGA
		Forward2: TCGCCAGCCTGTTTCCGCTTGGGTCGA
		Reverse2: ATGCTCGAGCTACAGGTCCTCCTCCGAGATCAACTTTTGTTCGCACATAGATATGGTGACGTTAATG
	Siah2lDRING MYC	Forward1: CATATCGATATGAGCCGTCCGTCCTCTGCG
		Reverse1: TCGGCGTGAGAGGAAACAGCGCTGTCA
		Forward2: TGACAGCGCTGTTTCCTCTCACGCCGA
		Reverse2: ATGCTCGAGTTACAGGTCCTCCTCCGAGATCAACTTTTGTTCGCACATAGAGATGGTCACGTTG
	GFP-NxN	Forward: CATGGATCCATGGTGAGCAAGGGCGAGGAG
		Reverse: ATGCTCGAGTCAATTCATATTTGGGGCATTCGGCTTGTACAGCTCGTCCATGCC
	GFP-Nlz2	Forward: CATGGATCCATGGTGAGCAAGGGCGAGGAG
		Reverse: ATGCTCGAGTCATGGACTGACCGGGGTGGATGGCTTGTACAGCTCGTCCATGCC
**qPCR**	Pax2a	Forward: TGTGTCAAGCGCTTCCAATG
		Reverse: TTCTCTTTTCGCCGTTGGAG
	GAPDH	Forward: CCTTATGGCCATGTGTTGAATTGTT
		Reverse: AACAATTCAACACATGGCCATAAGG

Biology Open • Supplementary information
